# Indole trimers with antibacterial activity against Gram-positive organisms produced using combinatorial biocatalysis

**DOI:** 10.1186/s13568-015-0125-4

**Published:** 2015-06-26

**Authors:** Kevin McClay, Shahila Mehboob, Jerry Yu, Bernard D Santarsiero, Jiangping Deng, James L Cook, Hyunyoung Jeong, Michael E Johnson, Robert J Steffan

**Affiliations:** CB&I Federal Services, 17 Princess Rd, Lawrenceville, NJ 08648 USA; College of Pharmacy, University of Illinois at Chicago, 900 South Ashland Avenue, Chicago, IL 60607 USA; Division of Infectious Diseases, Loyola University Chicago, Maywood, IL 60153 USA; Edward Hines Jr. VA Hospital, Hines, IL 60141 USA

**Keywords:** Antibacterial, Drug discovery, Combinatorial biocatalysis, Antibiotic resistance, Indole, Tryptanthrin

## Abstract

The I100V isoform of toluene-4-monooxygenase was used to catalyze the oxidative polymerization of anthranil and various indoles under mildly acidic conditions, favoring the production of trimers. Compounds produced in sufficient yield were purified and tested for their ability to inhibit the growth of *B. anthracis*, *E. faecalis*, *L. monocytogenes*, *S. aureus*, and in some cases, *F. tularensis*. 15 of the compounds displayed promising antibacterial activity (MIC < 5 µg/ml) against one or more of the strains tested, with the best MIC values being <0.8 µg/ml. All of these compounds had good selectivity, showing minimal cytotoxicity towards HepG2 cells. The structure was solved for six of the compounds that could be crystallized, revealing that minimally two classes of indole based trimers were produced. One compound class produced was a group of substituted derivatives of the natural product 2,2-bis(3-indolyl) indoxyl. The other group of compounds identified was classified as tryptanthrin-like compounds, all having multi-ring pendant groups attached at position 11 of tryptanthrin. One compound of particular interest, SAB-J85, had a structure that suggests that any compound, with a ring structure that can be activated by an oxygenase, might serve as a substrate for combinatorial biocatalysis.

## Introduction

A number of Gram-positive bacteria cause, or have the potential to cause, significant morbidity and mortality. Among these are pathogens that are encountered in the broader community and in hospital settings [*Staphylococci* (Ziakas et al. 2014), *Streptococci* (Nygård et al. 2014), *Enterococci* (Rosenberg Goldstein et al. 2014), and *Clostridia* (Goudarzi et al. 2014)] as well as bacteria that have the potential for intentional and malicious dispersal [*Bacillus anthracis* and *Listeria monocytogenes* (NIAID: Biodefense and emerging infectious diseases 2015)]. Of particular concern are isolates of these organisms that have acquired resistance to previously effective antibacterial agents. In the USA alone, >2,000,000 people are infected with bacteria that are resistant to one or more antibiotics, resulting in 23,000 deaths/year (CDC threat report 2013). Another significant outcome associated with antibacterial resistant infections is the economic burden associated with them. The direct cost has been estimated to be as high as $20 billion/year, primarily due to the fact that treating antibacterial resistant infections requires more intensive treatments and longer, more costly hospital stays.

The incidence of infection by drug resistant bacterial pathogens varies by location, but overall, appears to be on the rise. For example, the prevalence of vancomycin resistant *Enterococcus* infections in Chinese hospitals went from 0% in 2005 to 4.9% in 2010, which is still relatively low, compared to the 18.9% prevalence rate found hospitals in Taiwan (Kang and Song 2013). A study conducted in the USA found that the prevalence of methicillin resistance in *Staphylococcus aureus* (MRSA) increased >3 fold between 2002 and 2007 and ultimately MRSA represented 51% of all *Staphylococcus* infections during the study period (Gerber et al. 2009). The observed MRSA prevalence in the USA falls roughly midway between the prevalence of MRSA infection in India (22.6%) and that found in Sri Lanka [86.5% (Kang and Song 2013)]. While prevalence rates are local, the threat of antibacterial resistant pathogens is a worldwide problem, as resistance that develops in one area can rapidly spread to other parts of the globe. This situation clearly requires the development of new antibacterial compounds to replace the ones that face dwindling efficacy. Though more challenging to produce, antibacterial compounds that have novel structures, mechanisms of action and/or exploit previously unrecognized targets, as compared to those that have already reached the market, are the most logical choices for combating drug resistant pathogens (Donadio et al. 2010; Gwynn et al. 2010). Despite the need for new antibiotics with new modes of action, there has been a “discovery void”, where no new antibiotics were discovered between 1987 and 2011 [though some drugs have been introduced to the market place, they were discovered much earlier (Silver 2011)].

Arguably the best source for new antibacterials has historically been natural products, but efforts to exploit natural products as a source for new antibacterials has been in decline for many years (Brown et al. 2014). There are a number of contributing factors that have been postulated to have caused this decline. Among these factors is the idea that the most abundant and effective antibacterials have already been discovered and their relative abundance masks and otherwise complicates the discovery of novel but more rare antibacterials (Brown et al. 2014). Another often cited problem is that the high-throughput screening (HTS) of chemical libraries against specific and essential bacterial enzymes has not been as successful as hoped. While both natural products and HTS approaches have obvious merits, the development of new discovery approaches would be beneficial.

Previously, a biocatalytic approach for creating a library of indole based compounds was described, that focused primarily on isolating compounds with antibacterial activity towards *Mycobacterium tuberculosis* (TB, McClay et al. 2013). While exploring <1% of the theoretical chemical space that can be accessed with the described approach, 22 compounds were isolated that had minimum inhibitory concentrations (MIC) against TB of <2 µg/ml, with the most promising compound, SAB-P1, having a MIC of 0.16 µg/ml. It was also noted that a number of indole trimers, with varying degrees of potency towards TB, also had antibacterial activity towards many Gram-positive bacteria, which might make them more useful than the TB specific dimers. In this study, to gain a better understanding of the range of the indole based trimers that can be produced using combinatorial biocatalysis, and to assess their potential application as antibacterials, fermentation conditions were altered to favor the production of indole trimers. For this study, the only catalyst used was the I100V isoform of the toluene-4-monooxygenase of *Pseudomonas mendocina* KR1 expressed in *E. coli* (McClay et al. 2013). The range of substrates provided to the catalyst was restricted to anthranil and selected derivatives of indole with pendant substitutions. The compounds produced in good yield were screened for activity against the Gram-positive representatives of the ESKAPE pathogens group [*Enterococcus faecalis* and *Staphylococcus aureus* (Boucher et al. 2009)] and some representatives of the National Institute of Allergy and Infectious Diseases (NIAID) priority pathogens group [*Bacillus anthracis*, *Listeria monocytogenes*, and *Francisella tularensis* (NIAID webpage)]. The HepG2 liver cell line was used to determine mammalian toxicity. A number of trimers had MIC values of <2 µg/ml against multiple pathogens and a broad therapeutic window based on HepG2 toxicity. X-ray crystallography was used to solve the structure of six of the trimers produced. Structural data showed that the trimers described could be categorized into two groups, those that were tryptanthrin-like and those there were related to the natural product 2,2-bis(3-indolyl) indoxyl (BII). Of particular interest was the antibacterial compound SAB-J85, which has a structure that suggests that any number of compounds with ring structures that can be oxidized by the I100V catalyst (or other oxygenases) could serve as useful substrates for combinatorial biocatalysis.

## Materials and methods

### Biotransformation conditions

The creation of the toluene-4-monooxygenase based biocatalyst (I100V), and the chemicals used have been described elsewhere (McClay et al. 2013). However, the fermentation conditions used were modified to favor the production of indole based trimers by lowering the pH of the media used for the biotransformation as described below. To prepare the biocatalyst for the biotransformation reactions, a 10-ml culture of *E. coli* carrying the I100V oxygenase was grown overnight in LB broth amended with 25 μg/ml kanamycin. The starter culture was aseptically transferred to a 3-l fermentor filled to 80% capacity with basal salts medium [BSM (Hareland et al. 1975)]. Glucose (2 g/l) and kanamycin (25 µg/ml) were added to the medium, which was stirred at 200 rpm, at 30°C, while maintaining the pH between 6.8 and 7.2 by the automated addition of sodium hydroxide. When the initial carbon source was consumed, as judged by a decrease in the rate of oxygen consumption, 2 g/l of glucose was added and the expression of I100V was induced by adding sodium benzoate to a final concentration of 50 mg/l, followed by 6–8 h of additional growth. The cells were harvested via centrifugation and suspended in 2 l of BSM which had been amended with HCl to bring the pH down from ~7.2 to 6–5.5. The cells were then distributed as 250-ml aliquots to baffled 2-l Erlenmeyer flasks. The biotransformations were started by adding a total of 20–30 mg of each substrate dissolved in 200–300 µl of ethanol along with 2 g/l of glucose and 50 mg/l of sodium benzoate. The biotransformation reactions were allowed to incubate overnight with shaking at 100 rpm at 30°C. The products of the biotransformation were harvested and purified as previously described (McClay et al. 2013).

### Determination of MIC and MBC

Minimum inhibitory concentration experiments were performed with four Gram-positive bacterial strains. These include *B. anthracis* −Δ ANR strain (plasmid-cured Ames strain of *B. anthracis*), *E. faecalis* (ATCC #29212), *L. monocytogenes* (ATCC #104035), *S. aureus* (ATCC #29213) and the Gram-negative *F. tularensis* (ATCC #43300). MIC values were determined by the microdilution method. LB media was added to each well in a row on a sterile 96-well flat bottom tissue culture plate. 96 µl of LB media was added to the first column and 50 µl was added to all subsequent wells. The compounds to be tested were added to the first column for a final well volume of 100 µl. Compounds were then serially diluted (2-fold) across the columns of wells by pipetting and mixing 50 µl of solution. The extra 50 µl was discarded from the final well. Ciprofloxacin was used as a control in these studies. Prior to setting up the MIC plates, the appropriate bacterial cultures were grown to mid log-phase and subsequently diluted to an OD_600_ = 0.004 with fresh LB media. 50 µl of this bacterial culture was added to each well of the plate, and the plate was then incubated at 37°C overnight without shaking. The MIC value for each compound represents the lowest concentration of the compound that resulted in a clear well with no signs of visible growth. To determine the minimum bactericidal concentration (MBC), aliquots from clear wells containing increasing concentrations of the compound were plated onto antibiotic-free agar plates, and the lowest concentration at which no visible growth was observed was reported as the MBC.

### Cytotoxicity

The cytotoxicity of the test compounds was determined using the sulforhodamine B (SRB) assay (Vichai and Kirtikara 2006) in the human hepatoma cell line HepG2 (ATCC #HB-8065). This assay directly measures cellular protein content. The HepG2 cells were seeded into 96 well plates and incubated for 24 h with different concentrations of each of the test compounds. At the end of incubation period, cells were fixed with 10% (wt/v) trichloroacetic acid and stained for 30 min, followed by removal of excess dye by washing with 1% (v/v) acetic acid. The protein-bound dye was dissolved in 10 mM Tris base, and absorbance was determined by using a spectrophotometer set at 510 nm. The results were compared to the results obtained with untreated cells. A dose–response curve was obtained and IC_50_ values determined.

### X-ray crystallography

The compounds, purified as previously described (McClay et al. 2013), were dissolved in a minimum volume of acetone, typically 2–3 ml in a 25-ml screw cap glass vial. Crystal growth trials were initially conducted with the cap off. If results were unsatisfactory, the cap was placed over the vial and tightened as needed to slow the evaporation rate down to enable the growth of larger crystals. X-ray diffraction data was collected at the Advanced Photon source LS-CAT beamline 21-ID-D using a using a MAR 300 CCD detector. The integration of intensities and refinement of unit cell parameters and orientation matrix were carried out with the HKL2000 (Otwinowski and Minor 1997) and XDS (Kabsch 2010) program packages. The structures were solved and refined using SHELX (Sheldrick 2007).

## Results

### Evaluation of biological activity

The four Gram-positive bacteria species, *B. anthracis* (Δ-ANR strain), *E. faecalis*, *L. monocytogenes*, and *S. aureus*, were challenged with 75 test compounds. 28 of these compounds displayed promising antibacterial activity (Table 1) with many of these compounds being active against more than one strain tested. Four compounds (SAB-J3, SAB-J45, SAB-J51, and SAB-J76) were active against all four strains tested. Additionally, SAB-J3 was tested against the Gram-negative organism *F. tularensis* and found to have a MIC of 1.56 µg/ml. All of the compounds that were active against more than one bacterial strain were assayed against HepG2 cells to determine their toxicity towards mammalian cells. Most of these compounds were not found to be significantly cytotoxic, displaying a selectivity index >10 fold ([cytotoxicity]/[MIC]). SAB-J3 and SAB-J45 had the most favorable selectivity index ranging between 64 and 128 fold for SAB-J3, and 16-64 fold for SAB-J45, depending on which bacterial species served as the target. As indicated in Table 1, some of the compounds were not fully soluble in aqueous media at the highest concentrations tested (100 µg/ml), thus they were not toxic at the limit of their solubility, which might differ from the number reported.

**Table 1:** MIC, MBC and IC50 values (µg/ml) for selected compounds

Test compounds	Substrate(s)	MIC/MBC				IC50
		*B. anthracis*	*E. faecalis*	*L. monocytogenes*	*S. aureus*	HepG2
Ciproflaxin	na	0.07	0.52	1.56	0.68	nd
Tryptanthrin	na	>25	nd	nd	4.6	nd
SAB-R1	Ind(Ant)	>12.5	>12.5	>12.5	>12.5	nd
SAB-C10	5C	3.12/12.5	12.5	12.5	6.25/25	>100
SAB-J2	5F	6.25	12.5	12.5	3.12/12.5	49.2
SAB-J3	5B	1.56/12.5	*0.78/>25*	*1.56/>25*	1.56/12.5	>100^a^
SAB-J7	5C(Ant)	6.25	>25	>25	>25	nd
SAB-J11	Ind(Ant)	3.12/25	>25	>25	6.25	>100
SAB-J16	5B	6.25	12.5	25	12.5	nd
SAB-J19	4B(7C)	6.25	>25	25	6.25	nd
SAB-J20	6C	6.25	>25	>25	6.25	nd
SAB-J22	5F(1M)	6.25	>25	>25	>25	nd
SAB-J25	6C(7C)	3.12/25	12.5	25	6.25	>100^a^
SAB-J35	4B	6.25	>25	>25	>25	nd
SAB-J36	6C	6.25	25	>25	12.5	nd
SAB-J37	5B(6C)	3.12/25	12.5	12.5	6.25	>100^a^
SAB-J38	5C(Ant)	3.12/12.5	>25	25	6.25	>100
SAB-J39	5C(Ant)	6.25	>25	>25	6.25	>100
SAB-J45	5B(Ant)	1.6/3.3	3.1	6.3	3.1/12.5	>100
SAB-7f-R1	7F(Ant)	>12.5	>12.5	>125	3.1/>12.5	nd
SAB-J51	5F(7F)	0.88/4.69	1.95/>12.5	1.76/>12.5	1.17/>12.5	78.4^a^
SAB-J62	5F(Ant)	5.47/>12.5	>12.5	>12.5	5.47/>12.5	>100
SAB-J63	5B(Ant)	3.12/>12.5	>12.5	>12.5	5.4/>12.5	>100^a^
SAB-J65	5C(Ant)	2.15/>12.5	>12.5	>12.5	*0.98/>12.5*	>100
SAB-J69	5B(Ant)	3.52/>12.5	>12.5	>12.5	3.12/>12.5	>100
SAB-J76	6C(Ant)	*0.78/2.34*	2.73/>12.5	7.29/>12.5	2.15/12.5	43.8
SAB-J78	6C(Ant)	1.56/>12.5	>12.5	>12.5	3.9/>12.5	>100
SAB-J79	6C(Ant)	0.78/4.69	>12.5	>12.5	1.37/9.38	>100
SAB-J81	5B(Ant)	>7.29	>12.5	>12.5	1.37/>12.5	>100
SAB-J82	5B(Ant)	6.25	>12.5	>12.5	6.25	nd
SAB-J85	6C(Ant)	0.78	nd	nd	3.13	nd

The most effective compound against each strain shown in italic font.

*na* not applicable, *nd* not determined.

Substrate abbreviations: *Ind* indole, *Ant* anthranil, *4B* 4-bromoindole, *5B* 5-bromoindole, *5f* 5-fluoroindole, *6F* 6-fluoroindole, *7F* 7-fluoroindole, *5C* 5-chloroindole, *6C* 6-chloroindole, *7C* 7-chloroindole, *1M* 1-methylindole.

^a^Compounds partially insoluble at concentration listed.

### Structural characterization

The structure of six of the indole based trimers, biocatalytically produced under mildly acid conditions, were solved using X-ray crystallography. Three of these, SAB-7fR1, SAB-J78, and SAB-J83 are merely substituted isomers of SAB-R1, which has been previously reported (McClay et al. 2013). The substitution pattern of these compounds directly reflects the substitution pattern of the substrates provided. Another substituted isomer of SAB-R1, SAB-R1-OH, contains an additional oxygen atom. The compound SAB-J85 is also structurally similar to SAB-R1, but, the pendant anthranil moiety is attached via a linkage between the six member ring of anthranil and C11 of tryptanthrin, as opposed the heterocyclic ring that was involved in all examples mentioned above. The structure of compound, SAB-J50, is comprised of two indoles and one 1-methylindole. Excluding the pendant methyl group, the structure of SAB-J50 is identical to that of 2,2-bis(3-indolyl) indoxyl (BII), which has been previously described (Veluri et al. 2003; Watsuji et al. 2007; Ganachaud et al. 2008).

## Discussion

**Figure 1:**
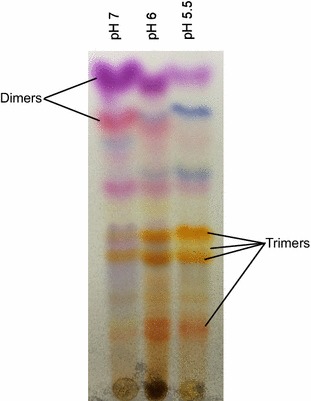
The effect decreased pH has on the product distribution during combinatorial biocatalysis. In each case the catalyst was the I100V isoform of T4MO and the two substrates were anthranil and 6-chloroindole. The pH values shown are the values for the media prior to adding cells and substrates, the pH at the end of the reactions was not measured.

To further explore the potential and utility of using combinatorial biocatalysis to create novel compounds with useful biological activity, fermentations were conducted under mildly acidic starting conditions (pH 6) to favor the production of indole based trimers (Figure 1). Previous work had shown that while indole based dimers produced using combinatorial biocatalysis were effective against *M. tuberculosis*, indole trimers had a broader spectrum of activity (McClay et al. 2013) and ultimately might be more useful. The trimers characterized here fell into two broad categories, those with a tryptanthrin-like structure and those with a 2,2-bis(3-indolyl) indoxyl (BII) like structure.

**Figure 2:**
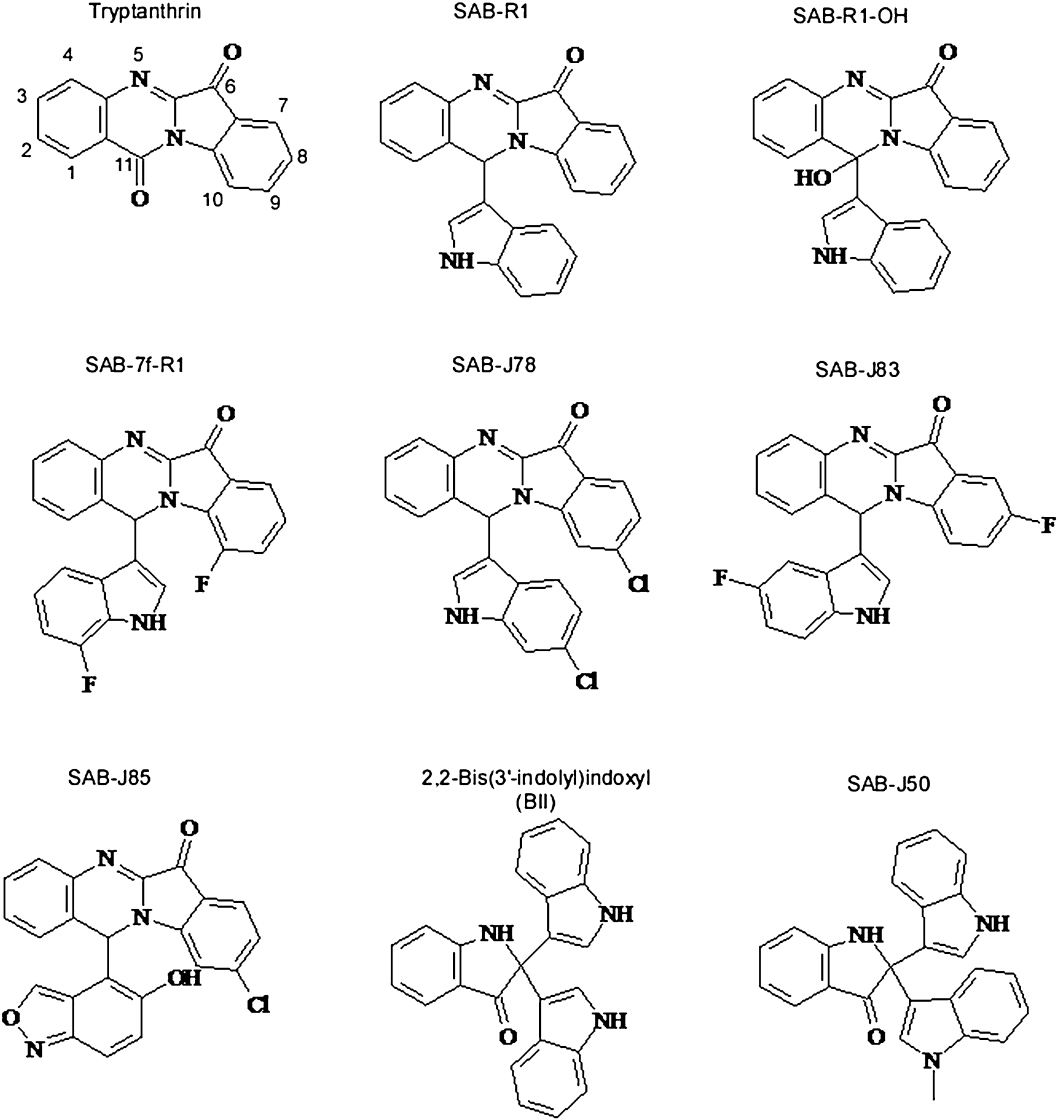
Structure of key compounds mentioned in text.

Tryptanthrin (Figure 2) is an intriguing molecule that holds promise for treating a number of disease states including cancer (Liao et al. 2013; Chan et al. 2009; Jao et al. 2008), malaria (Bhattacharjee et al. 2002), sleeping sickness (Bakshi et al. 2009), toxoplasmosis (Krivogorsky et al. 2008), and leishmaniasis (Bhattacharjee et al. 2002). Furthermore, studies have shown that certain tryptanthrin derivatives are effective antibacterial agents against both *E. coli* (Bandekar et al. 2010) and *M. tuberculosis* (Hwang et al. 2013) in vitro. In previous studies the most potent derivatives of tryptanthrin active against *E. coli*, TB, malaria, trypanosomiasis, and toxoplasmosis have pendant halo- or nitro substitutions at position 8 of tryptanthrin [though other substitution can also be present (Bandekar et al. 2010; Hwang et al. 2013; Scovill et al. 2002; Krivogorsky et al. 2008)]. A mechanism of action for tryptanthrin and its derivatives has not been firmly established though an in silico study suggested that tryptanthrins might function as inhibitors of the enoyl-acyl carrier protein reductase InhA (Tripathi et al. 2012). It is worth noting that in vivo, tryptanthrins failed to reduce the *M. tuberculosis* burden in mice, perhaps due to poor absorption and/or rapid elimination (Hwang et al. 2013).

In this study, a new class of tryptanthrin-like compounds was isolated and identified. All of the tryptanthrin-like compounds for which a structure was determined shared two characteristics. First, they all had pendant substitutions at position number 11 (Figure 2). Second, they all had an intense red/orange color easily visible on TLC plates. The first compound of this class that was isolated was, SAB-R1, which was previously described in McClay et al. (2013). SAB-R1, derived from un-substituted indole and anthranil, did not have appreciable antibacterial activity against the four organisms tested here (MIC > 25 µg/ml) or Mtb. However, isomers of SAB-R1 derived from indole isomers with various halogen substitutions around the aromatic ring have a much higher degree of antibacterial activity, in line with previous studies mentioned above where various substitutions at position 8 resulted in more efficacious compounds. Both the position and size of the substituting halogen seem to impact both the MIC and the spectrum of activity of the antibacterial compounds. The SAB-R1 derivatives that resulted from combining anthranil with 5-fluoroindole (SAB-J83) and 7-fluoroindole (SAB-7f-R1) had better MIC values than SAB-R1, but SAB-7f-R1 was ~4 times as effective against *S. aureus* (MIC = 3.1 µg/ml) as was the SAB-J83 (Table 1). The compound derived from 6-chloroindole (SAB-J78) was equally effective against *S. aureus* as was SAB-7f-R1, but was more effective against *B. anthracis* (MIC = 1.56 µg/ml). Finally, SAB-J45, the putative SAB-R1 isomer derived from 5-bromoindole (based on TLC and LC/MS analysis in the absence of crystal structure data) was effective against all four organisms tested here with MIC values ranging from 1.6 to 6.3 µg/ml.

Molecular weight analysis indicated that many of the orange compounds that migrated more slowly during TLC separation had molecular weights consistent with them being SAB-R1 derivatives carrying an additional oxygen atom. The crystal structures of SAB-R1-OH and SAB-J85 show that the additional oxygen groups can be attached either directly to carbon 11 of the tryptanthrin portion of the compound (SAB-R1-OH, which was not tested for antibacterial activity because of limited supply), or indirectly via the pendant group as is the case with SAB-J85. The compound SAB-J85 is a particularly interesting compound that resulted from the polymerization of 2 anthranils and 1 indole. Unlike all of the other tryptanthrin-like compounds described here, which are linked via the highly reactive C3 of indole and the C11 of tryptanthrin, SAB-J85 is linked via the comparatively inert 6-member ring of anthranil. It is of interest to note that the two compounds, SAB-J85 and the more hydrophobic compound SAB-J78, differ only in the makeup of their pendant groups, yet they have very similar MIC values for the two organisms that were challenged with both compounds. This implies that the antibacterial activity of these compounds might be in part or in whole associated with the tryptanthrin portion of the molecule. If such is the case, the entirety of the pendant group might be a substrate for further modifications that could impact the absorption and distribution of the compounds.

In addition to the tryptanthrin-like compounds mentioned above, the other group of antibacterial indole trimers described in this study are believed to be analogs of the compound BII (Figure 2). However, most of these putative BII analogs did not crystallize under the conditions described, making X-ray crystallography impossible. The conclusion that they have the same core structure as BII is based on a number of lines of evidence. First, it has been previously shown that BII can be formed by a laccase catalyzed oxidation of indole (Ganachaud et al. 2008). Second, the crystal structure of SAB-J50 (Figure 2), is identical to BII, excluding the pendant methyl group. Finally, all of the other putative representatives of this compound class had molecular weights, spectral and fluorescent characteristics, and TLC migration patterns that were consistent with being of the same chemotype as SAB-J50. BII is a natural product produced by the *Bacillus* symbiont, *Symbiobacterium thermophilum* (Watsuji et al. 2007) and functions as a reversible growth inhibitor of both the Gram-negative *S. thermophilum* (MIC = 12 µg/ml) and Gram-positive *Rhodococcus* sp. strain RHA1 (MIC = 4 μg/ml), but was not effective against 12 other bacterial species at >14 µg/ml, including *B. subtilis* 168. Previously, SAB-C10, the presumptive isomer of BII derived from 5-chloroindole, was described and it was noted that SAB-C10 was effective against *B. subtilis* 168 (as well as *B. cereus*, *E. faecalis*, *M. smegmatis*, *S. epidermidis*, and *C. acetobutylicum*) at <4 µg/ml (McClay et al. 2013). This work expanded on those findings creating new fluoro-, chloro-, and bromo-derivatives of BII (SAB-J2, -J3, -J51, and -J76), which along with SAB-C10 were tested against the suite of Gram-positive organisms (Table 1). The 5-bromo-derivative of BII (SAB-J3) had the best activity profile inhibiting all of the pathogens at 1.56 µg/ml or below, while having no observable cytotoxicity for HepG2 cells at the limits of its solubility. Two other derivatives of BII, SAB-J51, (derived from a mixture of 5-chloro- and 7-chloroindole) and SAB-J76 (derived from 6-chloroindole) had comparable MIC values to SAB-J3. However, SAB-J51 proved to be less soluble than SAB-J3 and SAB-J76 appeared to be more cytotoxic for HepG2 cells. Although not all possible halogenated derivatives of BII have been isolated and tested, it appears that as is the case with the tryptanthrin-like compounds, incorporation of larger halogens appears to increase the antibacterial activity, though positional effects are important too.

The data presented here further demonstrate that combinatorial biocatalysis is a useful tool for generating novel compounds with potentially useful biological activity. In this case novel derivatives of tryptanthrin-like and BII-like compounds with significant antibacterial activity were created, with the halogenated derivatives being superior to the non-halogenated compounds both in activity spectrum and in vitro efficacy. One of the more interesting compounds characterized here was the BII derivative produced from 3 5-bromoindoles (SAB-J3), which consistently had among the lowest MIC values with all of the Gram-positive organism tested and was also effective against the Gram-negative *F. tularensis* (MIC = 1.56 µg/ml). While the existence of unsubstituted BII and its antibacterial properties towards both Gram-positive and Gram-negative organisms have been reported periodically (Bell et al. 1994; Ganachaud et al. 2008; Sheinkman et al. 1978; Stull et al. 1995; Watsuji et al. 2007) to the best of our knowledge, this is the first report of the synthesis of substituted derivatives of BII and their improved antibacterial properties. Another interesting compound described herein is SAB-J85. The synthesis of SAB-J85, with the linkage of the six member ring of anthranil to position 11 of tryptanthrin suggests that any number of precursors with ring structures that can be activated by oxygenases could be used to create new tryptanthrin-like compounds. As a consequence of the oxygenase catalyzed activation, the new derivatives would likely also have a free hydroxyl group which would serve as a convenient site for further modification to help influence the absorption, metabolism, distribution, and excretion characteristic of the therapeutic compound. Tryptanthrin, BII, and improved derivatives thereof have not been distributed on a commercial scale for application as antibacterial agents, and thus, they might represent attractive scaffolds for developing new antibacterials.
